# Association of Structural Maintenance of Chromosome-1A Phosphorylation with Progression of Breast Cancer

**DOI:** 10.3390/cells14020128

**Published:** 2025-01-17

**Authors:** Sushma Yadav, Claudia M. Kowolik, Daniel Schmolze, Yuan Yuan, Min Lin, Arthur D. Riggs, David A. Horne

**Affiliations:** 1 Department of Cancer Biology and Molecular Medicine, City of Hope National Medical Center, Duarte, CA 91010, USA; ckowolik@coh.org (C.M.K.); mlin@coh.org (M.L.); 2 Diabetes and Metabolism Research Institute, City of Hope National Medical Center, Duarte, CA 91010, USA; ariggs@coh.org; 3 Department of Pathology, City of Hope National Medical Center, Duarte, CA 91010, USA; dschmolze@coh.org; 4 Breast Oncology, Cedars-Sinai Cancer Medical Center, Los Angeles, CA 90048, USA

**Keywords:** structural maintenance of chromosome-1A, phosphorylation, tumor progression, metastasis, breast cancer, immunohistochemistry

## Abstract

Structural maintenance of chromosome-1A (SMC1A) is overexpressed in various malignancies including triple-negative breast cancer (TNBC). As a core component of the cohesin complex, SMC1A was initially recognized for its involvement in chromosomal cohesion and DNA-repair pathways. However, recent studies have unveiled its pivotal role in epithelial–mesenchymal transition (EMT), metastasis, and chemo- and radio-resistance in cancer cells. In hepatocellular carcinoma, aberrant phosphorylation of SMC1A has been associated with enhanced cell proliferation and migration. Despite these insights, the precise role of SMC1A phosphorylation in breast cancer remains largely unexplored. This study represents the first investigation to test the phosphorylation status and subcellular localization of SMC1A (p-SMC1A) in breast cancer and normal breast tissues. Immunohistochemical (IHC) staining was conducted using previously validated phospho-SMC1A antibodies on a histological section and tissue microarray (TMA) comprising samples from primary, invasive, and metastatic breast cancer and normal breast tissues. Our results revealed that p-SMC1A staining intensity was lower in normal breast tissues compared to invasive or metastatic breast cancer tissues (*p* < 0.001). Approximately 40% of breast cancer tissue exhibited cytoplasmic/membranous localization of p-SMC1A, whereas nuclear expression was observed in normal breast tissues. Moreover, elevated phosphorylation levels were significantly associated with higher tumor grade and metastasis.

## 1. Introduction

Breast cancers remain one of the most prevalent malignancies globally. According to US Breast Cancer Statistics, it is projected that in 2024, approximately 310,720 new cases of invasive breast cancer were diagnosed in women in the US, accompanied by 56,500 new cases of non-invasive (in situ) breast cancer [1]. Prognostic and predictive factors, such as estrogen/progesterone receptor (ER/PR) status and HER-2/neu gene amplification, serve as effective therapeutic targets for hormonal therapy in breast cancer patients [2,3]. However, triple-negative breast cancer (TNBC), constituting 15–20% of diagnosed breast cancers, carries a worse prognosis than other subtypes due to the absence of HER-2/neu and ER/PR receptors [2,3,4,5]. Despite recent incorporation of immune checkpoint inhibitor and drug–antibody conjugates, prognosis of TNBC remains challenging due to the presence of intrinsic or acquired resistance [6,7,8,9]. Therefore, prioritizing the understanding of the molecular drivers of cancer progression and exploring diagnostic or predictive approaches remain crucial for this patient cohort.

Approximately 10–15% of TNBC cases carry BRCA1/2 mutation, and the BRCA proteins are crucial for DNA double-strand break repair and genomic stability maintenance [10,11,12,13,14]. The interaction between BRCA1 and structural maintenance of chromosomes-1A (SMC1A) likely contributes to BRCA1’s role in genomic stability [15,16,17]. SMC1A is part of the cohesin complex, along with SMC3, Rad21, and SA1/2, originally identified for its role in sister chromatid cohesion and DNA repair but now recognized for its involvement in cell proliferation and pluripotency maintenance [18,19,20,21,22,23,24,25,26]. Emerging evidence indicates the differential expression and function of cohesin in various cancers, including breast, colon, lung, glioma, colorectal, and prostate [27,28,29,30,31,32,33]. Our published findings demonstrated the differential overexpression, localization and function of SMC1A in TNBC cell proliferation and metastasis [27]. Moreover, loss of SMC1A function, achieved through SMC1A antisense, sensitized TNBCs to PARP inhibitors [27]. We have shown for the first time the role of SMC1A in radio-resistance and regulating epithelial–mesenchymal transition (EMT) and cancer stem-like properties in prostate cancer cells [33]. Other labs have illustrated its role in tumor progression and metastasis in various cancers [25,28,29,30,31,32].

SMC1A, a substrate of ataxia-telangiectasia mutated (ATM) kinase, undergoes phosphorylation at Serine 957 and Serine 966 post-ionizing radiation [16]. It is believed to be a downstream effector of the ATM-NBS1-BRCA pathway crucial for cell survival and chromosomal stability maintenance following DNA damage [16,17]. Additionally, SMC1A is phosphorylated by ATR in response to various stressors such as hypoxia, UV radiation, and hydroxyurea treatment [34]. In hepatocellular carcinoma, SMC1A is aberrantly phosphorylated at Serine 966, and its phosphorylation has been shown to promote cell proliferation and migration [35]. This suggests that SMC1A phosphorylation may play a pivotal role in cancer progression; however, its significance in breast cancer remains uncertain.

In this study, we assessed the expression and cellular localization of phosphorylated SMC1A protein (p-SMC1A) through immunohistochemical (IHC) staining utilizing histological sections of breast tumors and breast cancer tissue array, using the anti-SMC1A (phospho-S966) antibody previously validated for breast tumors. Expression and subcellular localization of p-SMC1A were analyzed using the VENTANA image viewer, and H-score was computed. Correlations between p-SMC1A expression, subcellular localization, and clinicopathological characteristics such as cancer stage, histological grade, and subtypes were examined. High levels of phosphorylation on SMC1A were associated with advanced tumor grade and negative hormone receptor status (estrogen receptor, progesterone receptor, and HER2).

Expression and cellular localization of p-SMC1A was also tested in a panel of breast cancer and normal epithelial cells by immunocytochemistry (ICC), flow cytometry and on-cell Western analysis. We also tested the expression of p-SMC1A protein in membranous, cytoplasmic, and nuclear fractions of breast cancer and normal epithelial cells by cellular fractionation and Western blot. Our results showed that SMC1A was aberrantly phosphorylated in breast cancer cells, and the phosphorylated protein mislocalized to the cytoplasm and membrane of selected cancer cells and not the normal cells. Our results support the hypothesis that phosphorylation of SMC1A may provide a predictive biomarker and potential therapeutic target for breast cancer tumors.

## 2. Materials and Methods

### 2.1. Reagents

Antibodies were purchased from Bethyl Labs, FORTIS Life Sciences (Houston, TX, USA) and Cell Signaling Technology (Beverly, MA, USA). The details of antibodies are given in Supplementary Table S1. DyLight^®^ 488- and 550-conjugated Goat anti-Rabbit IgGs were obtained from Bethyl Labs, FORTIS Life Sciences (Houston, TX, USA). Horseradish peroxidase (HRP)-conjugated anti-Mouse and anti-Rabbit secondary antibodies were procured from Thermo Fisher Scientific (Waltham, MA, USA). CellTag 700 Stain and IRDye^®^ 800CW Goat anti-Rabbit secondary antibody were from LICOR Biotechnology (Lincoln, NE, USA) The subcellular protein fractionation kit was from Thermo Fisher Scientific (Rockford, IL, USA). Sources of other reagents were the same as previously described [33].

### 2.2. Histological Tumor Tissue Samples and Tissue Microarray (TMA)

Breast cancer progression tissue array (TMA) was obtained from US Biomax (Derwood, MD, USA). Formalin-fixed, paraffin-embedded (FFPE) TMA slides (5 µm) were accompanied by age of the patient and tumor characteristics including histology, tumor grade, clinical stage, and expression of hormone receptors (ER, PR, HER2). Formalin-fixed paraffin-embedded tissues from TNBC patients, obtained from Pathology Biorepository, City of Hope, were processed, and serial sections of 5 μm thickness were prepared by Pathology core, City of Hope.

### 2.3. Cell Lines and Cultures

Breast cancer cell lines MCF10A, MCF7, SK-Br3, MDA-MB-231, MDA-MB-436, MDA-MB-468 were obtained from American Type Culture Collection (ATCC) (Manassas, VA, USA). Normal epithelial cells (HPNE) were from Lonza Bioscience (Morristown, NJ, USA). Cells were cultured at 37 °C with 5% CO_2_ in the following media: RPMI-1640 (MCF7), McCoy’s 5A (SK-BR-3), Leibovitz’s L-15 (MDA-MB-436), DMEM (MDA-MB-231, MDA-MB-468, HPNE), DMEM/F12 containing cholera toxin (0.1 µg/mL), insulin (10 µg/mL), hydrocortisone (0.5 µg/mL) and EGF (20 ng/mL) (MCF10A) supplemented with 10% fetal bovine serum (FBS). Cells were grown in tissue-culture-treated culture dishes, multi-well plates or chamber slides for assays.

### 2.4. Immunohistochemistry (IHC)

Immunohistochemical (IHC) staining was performed at the City of Hope Pathology Core Facility. The protocol was optimized by testing different sources and dilutions of phospho-SMC1A (S966) primary antibodies and methods of antigen retrieval. Validated p-SMC1A antibody from Bethyl labs was used for the IHC staining. Serial sections from the tumor tissues and tissue microarrays (5 μm) were deparaffinized in xylene, rehydrated in graded alcohol and then quenched in 3% hydrogen peroxide as described [33]. For heat-induced epitope retrieval, the sections were steamed with DIVA buffer (BioCare Medical) for 20 min. Slides were blocked for 5 min using Protein Block from Dako, and then, the slides were incubated with p-SMC1A antibody (1 mg/mL stock; 1:500 dilution) overnight at 4 °C. The next day, the slides were run on a Dako Auto stainer with a rabbit polymer from Dako, the EnVision+ horseradish peroxidase system for detection of rabbit primary antibodies. After washing in Dako buffer, slides were incubated with the chromogen diaminobenzidine tetrahydrochloride (DAB), counterstained with hematoxylin, and mounted. Positive and negative controls were included in each assay series.

### 2.5. Evaluation of Staining

Stained slides were scanned with the Ventana image viewer system (Ventana Medical Systems Inc., Oro Valley, AZ, USA). Expression of pSMC1A was evaluated in tissue compartments (epithelium vs. stromal) and subcellular compartments (nucleus, cytoplasm and membrane) in each core image by a trained pathologist using manual editing. p-SMC1A expression was evaluated by H-score, a product of the percentage of cells (0–100%) in each intensity category (0, 1+, 2+ and 3+). The final score is on a continuous scale between 0 and 300.

### 2.6. Immunocytochemistry (ICC)

Cellular localization of p-SMC1A was performed on a panel of breast cancer cells by a method described previously with slight modifications [27,33]. Briefly, cells (~20,000 cells/well) were grown in 15 µ-slide with 8 wells (Ibidi) in standard culture conditions. After 24 h, cells were washed with phosphate-buffered saline (PBS) and fixed with ice-cold methanol and acetic acid solution (7:1) followed by washing 3 times with PBS containing 0.05% Tween 20 (PBST). Nonspecific antibody interactions were minimized by incubating cells with 2% bovine serum albumin (BSA) in PBST for 60 min at room temperature. Subsequently, cells were incubated with anti-pSMC1A antibody (S966) (1 mg/mL stock; 1:500 dilution in PBST containing 2% BSA) overnight at 4 °C in a humidified chamber. After washing with PBST five times, the cells were incubated in DyLight^®^ 550-conjugated Goat anti-Rabbit IgG (1:500 dilution in PBST containing 2% BSA) for 1 h at room temperature in a humidified chamber, followed by washing with PBST five times. DAPI (4′,6-Diamidino-2-phenylindole) was used as a nuclear counterstain. Slides were analyzed by fluorescence microscope (Zeiss observer II), and the images were acquired at 40× resolution.

### 2.7. Cell Surface Localization of p-SMC1A by Flow Cytometry (FACS)

Surface localization of SMC1A was performed using indirect flow cytometry protocol as described [27]. Briefly, breast cancer (MDA-MB-231) and normal epithelial cells (MCF10a, HPNE) were harvested, washed with PBS and suspended in approximately 1 × 10^6^ cells/mL in ice-cold PBS, containing 10% FBS. Cells were incubated with anti-SMC1A or anti-pSMC1A antibody (S966) (1 mg/mL stock; 1:100 dilution) in 2% BSA/PBS on ice for 1 h, followed by washing with PBS and incubation with DyLight^®^ 488-conjugated secondary antibody for 30 min at 4 °C in dark. Cells were washed 5 times with PBS and resuspended in ice-cold PBS containing DAPI. Cells were captured by Miltenyi MACSQuant Analyzer 16 Flow Cytometer (Miltenyi Biotec, Inc., Auburn, CA, USA) and analyzed by FlowJo™ Software 10.7.1 gated on single cells followed by gating on DAPI negative cells.

### 2.8. Cell Surface Localization of p-SMC1A by On-Cell Western (OCW) Assay (LICOR)

For the on-cell Western (OCW) assay, breast cancer cells (MDA-MB-231 and MCF7) were plated in a 96-well plate (5000 cells/well) and grown overnight in a humidified cell culture incubator at 37 °C. Cells were blocked with 2% BSA and incubated with control IgG, anti-SMC1A IgG or anti-pSMC1A (phospho-S966) (1 mg/mL stock; 1:2000) for 1 h at 37 °C. Cells were then washed three times with cell culture media. Secondary antibody was then added (IRDye^®^ 800CW, Goat anti-Rabbit IgG) at a 1:1000 dilution for 1 h. Cells were washed twice with PBS, and 200 μL PBS was added to each well for visualization. The plate was visualized using a LI-COR Odyssey plate reader. To correct background signals not related to specific channel staining, the mean intensity of the wells incubated with control IgG was subtracted from the intensity of the wells incubated with primary antibodies. Cells were stained with CellTag 700 Stain for the cell normalization.

### 2.9. Cellular Fractionation and Western Blot Analysis

Subcellular distribution of pSMC1A was determined in a panel of breast cancer and normal epithelial cells using subcellular protein fractionation kit (Thermo Scientific) following manufacturer’s directions. Briefly, about 1 × 10^6^ cells grown in standard culture were harvested with trypsin-EDTA and then centrifuged at 500× *g* for 5 min and washed with ice-cold PBS. The cell pellet was resuspended in 0.2 mL CEB and incubated for 10 min at 4 °C with gentle shaking and was centrifuged at 500× *g* for 5 min to separate the cytoplasmic fraction from the pellet (cytoplasmic extract, CE). The pellet was resuspended in MEB containing protease inhibitors, vortexed and incubated at 4 °C for 10 min and centrifuged at 3000× *g* for 10 min to isolate plasma membrane in the pellet (membranous extract, ME). The pellet was dissolved in ice-cold NEB-containing protease inhibitor for 10 min, and the nuclear extract was collected (NE). Total protein extract was obtained as previously described [33]. Briefly, cells were lysed using RIPA buffer (Cell signaling, Danvers, MA, USA) supplemented with Halt Protease inhibitor cocktail (ThermoFisher). The protein concentration was quantified using the Pierce BCA Protein Assay Kit (Thermo Fisher Scientific), and appropriate amounts of lysates (~20 µg protein) were resolved over Criterion™ TGX precast gels and then transferred onto polyvinylidenedifluoride (PDVF) membrane. The blots were blocked using 5% BSA in Tris-buffered saline containing 0.1% Tween 20 (TBST) for 2 h at room temperature and probed using appropriate primary antibodies in 2% BSA in TBST buffer overnight at 4 °C. The details of antibodies are given in Supplementary Table S1. The membranes were then incubated with appropriate horseradish peroxidase-conjugated secondary antibody for 1 h at room temperature, washed with PBS, followed by detection using a chemiluminescence ECL kit (Bio-Rad, Hercules, CA, USA). Bands were visualized using the ChemiDoc Imaging System (Bio-Rad). Densitometry measurements of the bands in the Western blot of total cellular expression were performed using GS-900 calibrated densitometer (Bio-Rad) and normalized with GAPDH as an internal control.

### 2.10. Expression of SMC1A in Tumor and Normal Samples by Data Mining

The online analysis platform was used to explore the expression of SMC1A in invasive breast cancer and normal samples (from non-cancerous patients and pediatric tissues). This platform utilizes RNA-Seq data from The Cancer Genome Atlas (TCGA), Therapeutically Applicable Research to Generate Effective Treatments (TARGET), and The Genotype-Tissue Expression (GTEx) repositories. The altered expression within different platforms was analyzed separately, and statistical significance was computed using Mann–Whitney or Kruskal–Wallis tests. In this analysis, validation of differential expression was performed using equally sized training and test sets, which confirmed the reliability of the database in breast, colon, and lung cancer at a false discovery rate (FDR) below 10%. The online analysis platform enables unrestricted mining of the database and is accessible at TNMplot.com [36].

### 2.11. Statistical Analysis

Our primary outcome variables were the stage at diagnosis and histologic grade. Stage at diagnosis was categorized using the American Joint Committee on Cancer (AJCC) categories of 0, 1, 2, 3 and 4 [37]. Later stage at diagnosis was defined as stage 2, 3, 4 vs. early stage (0, 1). Histologic grade was assigned as low, intermediate, and high, and categorized as intermediate and high versus low for some analyses. Nuclear and cytoplasmic/membranous expression of p-SMC1A was evaluated as continuous scores (scale: 0–300). H-scores between the tumor and normal group were analyzed with a two-way protein expression analysis (immunohistochemical) using Student’s *t*-test. One-way ANOVA was then performed to determine and compare the mean for all the samples [38]. Adjusted *p*-values were obtained by employing a Tukey Honest Significant Differences test to compare mean of each pair. The *p*-value adjustment was selected to avoid overestimation of the H-scores’ mean differences among pairs. The statistical analyses were performed with the R software R-4.4.2 (https://www.r-project.org/, 7 September, 2019). Comparisons were significant when the adjusted or non-adjusted *p*-values were less than 0.05. All *p*-values are two-sided unless otherwise noted.

## 3. Results

### 3.1. Phosphorylation of SMC1A and Localization of pSMC1A in Normal and Tumor Tissues

**Figure 1:**
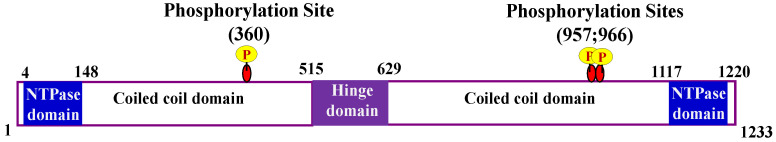
Schematic diagram for SMC1A showing the phosphorylation sites and domains of SMC1A. Phosphorylation sites include Ser360, Ser957 and Ser966. Phosphoserine S957 and S966 are shown to be phosphorylated by ATM and ATR [16,17,34]. Domains included coiled-coil domain, hinge-domain, and NTPase domains at N- and C-terminus of SMC1A.

SMC1A has three phosphorylation sites (Ser360, Ser957 and Ser966) (Figure 1) and is the major target substrate of ATM kinase [15,16]. Phosphorylated SMC1A has been shown to promote cell proliferation and migration in hepatocellular carcinoma [35].

**Figure 2:**
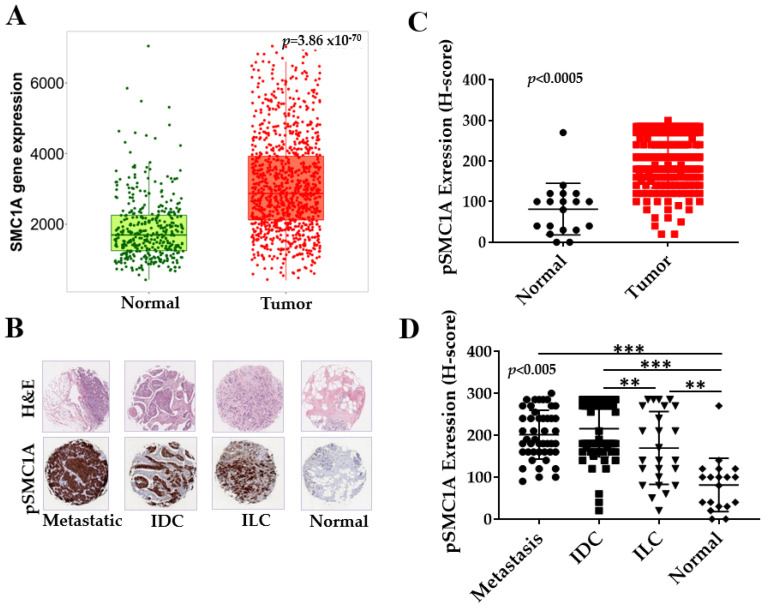
Expression and localization of pSMC1A in breast cancer progression. (**A**) Comparison of SMC1A expression in breast tumor and normal tissues by mining the integrated database shows significantly higher expression of SMC1A in breast tumor samples (*p* = 3.86 × 10^−70^). (**B**) Expression of phosphorylated SMC1A (Ser966) in breast cancer progression tissue microarray. H&E and immunohistochemical (IHC) staining of tissue microarray (TMA) of normal breast tissues, invasive ductal (IDC), invasive lobular (ILC), and metastatic carcinoma. IHC staining utilizes phospho-specific SMC1A antibody, p-SMC1A(Ser966). (**C**) Comparison of H-score representing the expression of pSMC1A in breast tumor and normal breast tissues (*p* < 0.0005). (**D**) Correlation of p-SMC1A expression in normal breast tissues, ILC, IDC and metastatic carcinoma. Difference in mean level of all four groups was calculated using one-way ANOVA (*p*-value < 0.005). ** denotes *p* values < 0.01 and *** denotes *p* values < 0.001.

Comparison of SMC1A expression in breast tumor and normal tissues was performed by mining the integrated database utilizing RNAseq data [36]. TPM count (Transcripts Per Million), a measure of gene expression level, was plotted. The data showed that SMC1A is significantly overexpressed in breast tumor tissues compared to normal samples (from non-cancerous patients and pediatric tissues) (Figure 2A). These results confirmed our previous work and the literature showing overexpression of SMC1A in various cancers [27,28,29,30,31,32,33,34,35,36]. To test the expression and localization of active SMC1A (pSMC1A) in breast tumors, we used histological sections of the breast cancer spectrum (from tissue microarray) and histological slides of TNBC sections including metastatic (lymph nodes or lung metastases originating from breast cancer), invasive ductal (IDC), lobular carcinoma (ILC) and normal or adjacent normal breast tissues. Each section was accompanied by H&E staining, grade, and stage of the tumor (Figure 2B). The clinical and pathological characteristics of the patients, from whom these samples were obtained, are summarized in Table 1.

To assess the subcellular expression of pSMC1A in tumor and normal cells, immunohistochemical staining was performed using hematoxylin as the background color and DAB to reveal positively stained tissue areas for pSMC1A protein [33]. Stained slides were scanned with the Ventana image viewer system (Ventana Medical Systems Inc., Oro Valley, AZ, USA). pSMC1A expression was evaluated by H-score, a product of the percentage of cells (0–100%) in each intensity category (0, 1+, 2+ and 3+). The final score presented is on a continuous scale between 0 and 300. Overall, expression of pSMC1A was higher in tumor than normal tissues (Figure 2C). The mean of normal tissues was 81.58 and for tumor tissues 201.5. Difference between means (tumor vs. normal) was 119.9 ± 16.26 with 95% confidence interval 152.0 to 87.88 and R squared (eta squared) value of 0.2156. Expression of pSMC1A was higher in invasive ductal (IDC) as compared to invasive lobular (ILC) and significantly higher than the normal tissues (Figure 2B,D). Positive correlation was observed between pSMC1A expression with the tumor progression (r = 0.95, *p* < 0.005). There was no significant difference in the expression of pSMC1A between metastatic tumor and IDC potentially because the metastatic tumor sections were not necessarily from the same patients as the primary tumor sections. Overall, SMC1A is aberrantly phosphorylated in breast tumors as compared to normal or adjacent normal tissues (Figure 2B–D). In normal breast tissue samples, the basal level of pSMC1A was either null or low, predominantly localized in the nucleus (Figure 2B–D).

**Figure 3:**
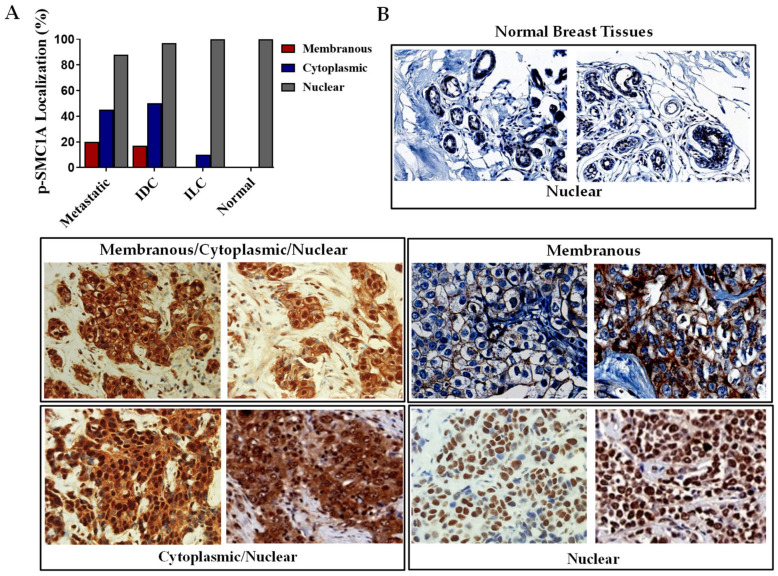
pSMC1A expression and cellular localization in breast tumor progression and normal breast tissues. (**A**) Percentages of cases positive for membrane, cytoplasmic and nuclear expression of pSMC1A in breast tumor and normal breast tissues. (**B**) Representative images of cellular localization of pSMC1A in breast tumor and normal tissues.

To explore the potential relationship of pSMC1A mislocalization with the progression of tumors, we also investigated the subcellular expression of pSMC1A in the histological sections of breast cancer spectrum and slides as described above (Table 1). In tumor tissues, significant inter-tumoral heterogeneity was observed in the patterns of membranous, cytoplasmic, and nuclear pSMC1A staining (Table 2). We observed a notable trend of higher level of membranous and cytosolic expression of pSMC1A in the metastatic tumor sections and IDC as compared with ILC and normal tissues (Figure 3A). Representative staining patterns of nuclear, membranous, and cytosolic pSMC1A are depicted in Figure 3B. Our results showed membranous expression of pSMC1A in 21% of metastatic tumors, 20% of IDC and 0% in ILC and normal breast tissues (Figure 3A and Table 2). pSMC1A was mislocalized to cytoplasm in 42% of metastatic tumors, in 49% of IDC tumors, and in 10% of ILC tumors (Figure 3A and Table 2). Normal breast tissues showed nuclear localization of pSMC1A (Figure 3A and Table 2).

### 3.2. Expression and Cellular Localization of pSMC1A in Breast Cancer and Normal Cells

**Figure 4:**
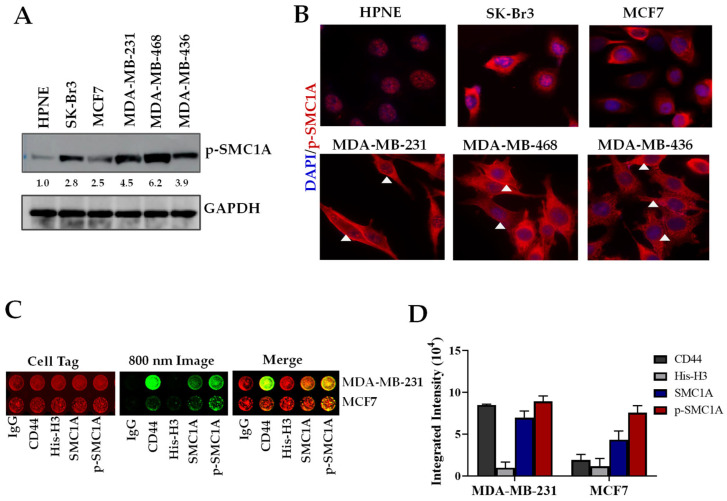
Cellular localization of pSMC1A in panel of breast cancer and normal cells. (**A**) pSMC1A expression was quantified by Western blot. Breast cancer cells have two- to five-fold-higher expression than normal epithelial cells. (**B**) Immunocytochemistry (ICC) shows heterogeneity of pSMC1A expression in cytoplasm, membrane and nucleus of breast cancer cells. White triangles shows the surface localization of pSMC1A. Normal epithelial cells have nuclear expression which is significantly lower than breast cancer cells. (**C**) Protein quantification by on-cell Western (LI-COR) showed localization of SMC1A and pSMC1A protein at the surface of cancer cells. (**D**) Integrated intensity of on-cell Western data shows higher surface expression in MDA-MB-231 compared to MCF7 cells.

The expression of pSMC1A was tested on a panel of breast cancer and normal epithelial cells by Western blot using p-SMC1A (S966) antibodies (1 mg/mL stock; 1:1000 dilution) as described in the Methods section. Densitometry measurements of the bands in Western blot showed there was two- to five-fold-higher expression of pSMC1A in breast cancer cells as compared to normal epithelial cells (Figure 4A). To test the cellular localization of p-SMC1A, immunocytochemistry was conducted using anti-pSMC1A (S966) antibodies on a panel of breast cancer cells and normal epithelial cells, cultured in 8-well chamber slides as described in the Methods section. Our results showed heterogeneous expression of pSMC1A amongst cancer cells (Figure 4B). In all breast cancer cells tested, pSMC1A was expressed in the nucleus, while there was heterogeneity in cytoplasmic and membranous expression. In a panel of TNBC cells (MDA-MB-436, MDA-MB-468 and MDA-MB-231), pSMC1A was present in the cytoplasm and membrane of cells. In hormone-dependent cell lines SK-Br3 and MCF7), pSMC1A showed membrane/cytoplasmic expression (Figure 4B). Normal epithelial cells (HPNE) showed significantly lower phosphorylation of SMC1A, and phosphorylated protein was expressed mainly in the nucleus (Figure 4B). Surface expression of pSMC1A was further tested in MDA-MB-231 and MCF7 cells by on-cell Western (OCW) assay. The integrated intensity plot showed that pSMC1A was present on the surface of MDA-MB-231 and MCF7 cells with higher intensity in MDA-MB-231 cells (Figure 4C,D). The mean of intensity for MDA-MB-231 was 6.334 and 3.745 for MCF7. Difference in mean intensity between MDA-MB-231 and MCF7 was 2.589 with SE of difference of 0.3814. CI of 95% of difference was 1.710 to 3.469. CD44 and His-H3 antibodies were used as positive and negative controls for surface expression

**Figure 5:**
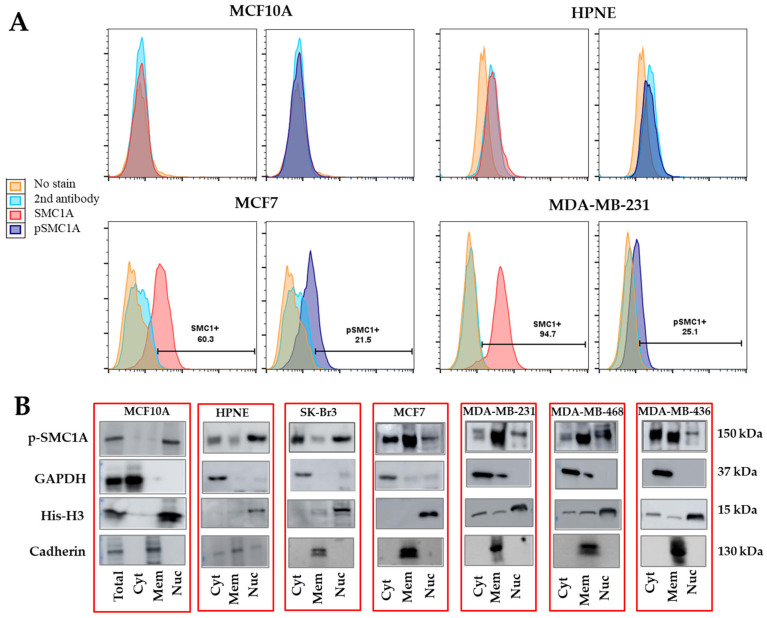
Subcellular localization of pSMC1A in panel of breast cancer and normal cells. (**A**) Surface localization of SMC1A and pSMC1A was performed using indirect flow cytometry protocol as described in the Methods section. Flow cytometry showed that SMC1A and pSMC1A were localized on the surface of cancer cells but not on the surface of normal cells. (**B**) Subcellular distribution of pSMC1A was determined in a panel of breast cancer cells using subcellular protein fractionation kit (Thermo Scientific) following manufacturer’s directions. Western blot results further confirmed pSMC1A expression on the surface of cancer cells but mainly nuclear expression in normal epithelial cells.

The membranous expression of pSMC1A was further tested on breast cancer and normal epithelial cells by flow cytometry using SMC1A and pSMC1A (Ser966) antibodies as described in the Methods section. Our results showed that both SMC1A and pSMC1A were expressed on the surface of breast cancer cells (MCF7, MDA-MB-231) but not on the normal cells (MCF10A, HPNE) (Figure 5A). Subcellular distribution of pSMC1A was also determined in a panel of breast cancer and normal cells using subcellular protein fractionation kit (Thermo Scientific) following manufacturer’s directions. Appropriate amounts of lysates (~20 µg protein) were resolved over Criterion™ TGX precast gels and transferred onto polyvinylidene difluoride (PDVF) membrane. The blots were probed using pSMC1A and other primary antibodies in 2% BSA in TBST buffer overnight at 4 °C. The details of antibodies are given in Supplementary Table S1. GAPDH, Cadherin (CDH1 or CDH2) and His-H3 were used as cytoplasmic, membrane and nuclear markers. Our results showed that there was heterogeneity in the localization of pSMC1A in the breast cancer cells. Normal epithelial cells (MCF10A and HPNE) showed pSMC1A expression predominantly in the nucleus, while in breast cancer cells, pSMC1A was present in membrane, cytoplasmic, and nuclear fractions (Figure 5B). Overall, these results showed that SMC1A was aberrantly phosphorylated in breast cancer cells and phosphorylated SMC1A mislocalized to the cytoplasm and membrane of some cancer cells.

## 4. Discussion

Breast cancer is the second most frequently diagnosed cancer worldwide. In 2024, an estimated 310,720 new cases of invasive breast cancer were diagnosed in women in the US, accompanied by 56,500 new cases of non-invasive (in situ) breast cancer [1]. Significant progress has been made in treating patients with estrogen receptor-positive (ER+) tumors in the last decade, since approximately 70–80% of these patients are considered to be ideal candidates for endocrine therapy alone or combination therapy [4,5]. Treatment of metastatic TNBC patients remains challenging despite recent incorporation of immune checkpoint inhibitors and antibody–drug conjugates targeting trop-2 (sacituzumab govitecan) or HER-2 (trastuzumab deruxtecan), largely due to tumor heterogeneity and acquired chemotherapy resistance [39,40]. Therefore, identification of new therapeutic targets and development of novel therapeutic strategies remain an unmet need and a high priority.

Structural maintenance of chromosome-1 A (SMC1A) is a core component of the cohesin complex, is traditionally considered a nuclear protein and known for its role in sister chromatid cohesion and chromosomal maintenance [20,21,22,23,24,41]. However, recent findings indicate its presence not only in the cytoplasm but also on the surface of cancer cells. Notably, SMC1A contains a positively charged amino terminus, rich in hydrophobic amino acids which may facilitate its membrane direction and lipid bilayer binding [42,43]. In addition, SMC1A interacts with proteins such as BRCA1 and Nup98, potentially regulating its localization in various cell types [44]. It is noteworthy, that Bamacan, a homolog of SMC3 (a binding partner of SMC1A) is found in basement membranes and cardiomyocytes, as well as in exosomes secreted from the breast tumors [45,46,47].

There is increasing evidence that SMC1A is closely associated with various cancer types, and our previous work has shown the role of SMC1A in the progression and metastasis of TNBC and metastatic castration-resistant prostate cancer cells [27,33]. In glioma, suppression of the SMC1A gene resulted in inhibition of tumor cell growth [30]. Overexpression of the SMC1A has been shown to be associated with tumor progression and poor prognosis [28,32]. In colorectal cancer, mutations and aberrant expression of SMC1A have been shown to be involved in tumor development [28]. In addition, SMC1A expression is significantly higher in carcinomas than in normal mucosa and early adenomas [31]. Overexpression of SMC1A was identified as a predictor of poor prognosis in late-stage colorectal cancer [31]. These findings suggest that overexpression of SMC1A may play a role in cancer pathogenesis.

SMC1A has been shown to be phosphorylated by ATM-kinase in response to x-irradiation and hypoxia/regeneration (H/R) and involved in cell survival, cell cycle regulation and DNA damage repair [16,17]. Moreover, SMC1A has shown to be aberrantly phosphorylated and involved in progression of hepatocellular carcinoma [35]. Given that phosphorylation of SMC1A is a crucial event in the regulation of diverse biological processes such as DNA damage repair, and tumorigenesis, we examined the expression and subcellular localization of phosphorylated SMC1A in human breast cancer progression, which included the primary as well as invasive and metastatic breast cancer tissues using IHC. Although we observed a lot of heterogeneity in the expression and localization of pSMC1A in breast cancer tissues, expression of pSMC1A was significantly higher in breast cancer compared to normal breast tissues. This is consistent with a previous study showing the role of SMC1A overexpression and phosphorylation in HCC progression [35]. While low to no phosphorylation of SMC1A was found on the surface of normal epithelial cells, we found aberrant phosphorylation of SMC1A on the surface of breast cancer cells known to have a high rate of proliferation. We found a positive correlation of SMC1A phosphorylation with tumor progression, suggesting a potential role of this molecule in the initiation and progression of breast cancer. Cytosolic/membrane expression of pSMC1A was observed at the metastatic sites and ductal and lobular carcinoma, while only nuclear expression was found in normal breast tissues. However, the cause of mislocalization of SMC1A and pSMC1A needs to be further investigated. The potential role of SMC1A localization and phosphorylation in radiation resistance and EMT will be explored in our future studies. Our current results support the potential role of SMC1A phosphorylation in breast cancer progression, and p-SMC1A may have prognostic value in breast cancer patients.

## 5. Patents

A patent related to this work was filed by City of Hope.

## Figures and Tables

**Table 1:** Characteristics of breast cancer progression tissue array and TNBC patient’s cohort.

Breast Cancer Progression Tissue Array and TNBC Patients Cohort Characteristics	N	%
**Tissue Array; Age at diagnosis (N = 161)**		
<50	99	61
50+	62	39
**Stage at diagnosis**		
Early (IA, IB)	31	19
Late (IIA, B, IIIA, IIIB)	63	39
Metastatic	48	29
Unknown	19	12
**Histologic Grade**		
Low/Intermediate	35	21
High	81	50
Unknown	45	29
**Histology**		
Metastatic	48	22
Invasive ductal (IDC)	69	48
Invasive lobular (ILC)	21	15
Squamous cell	4	3
Normal/Other	19	13
**Molecular subtype**		
TNBC	59	47
HER2 rich	33	26
Other	34	27
**TNBC patient samples from COH**		
**Age at diagnosis (N = 26)**		
<50	10	38
50+	16	61
Stage at diagnosis		
Early (IA, IB)	6	23
Late (IIA, IIB, IIIA, IIIB)	13	50
Metastatic	5	19
Unknown	2	7
**Histologic Grade**		
Low/Intermediate	3	10
High	15	57
Unknown	8	31
**Histology**		
Metastatic	5	19
Invasive ductal (IDC)	20	78
Invasive lobular (ILC)	0	0
Squamous cell	0	0
Normal/Other	1	4
**Molecular subtype**		
TNBC	26	100
HER2 rich	0	0
Other	0	0

**Table 2:** Summary of membranous, cytoplasmic, and nuclear expression of p-SMC1A in normal vs. invasive breast tissues.

p-SMC1A Localization in Normal vs. Breast Tumor Tissue.				
		Membranous Score	Cytoplasmic Score	Nuclear Score
Variable	N	# Cases %	# Cases %	# Cases %
Metastatic	53	11 21	22 42	47 89
Invasive Ductal (IDC)	89	18 20	44 49	86 97
Invasive Lobular (ILC)	21	0 0	2 10	21 100
Squamous Cell	4	0 0	3 75	4 100
Adjacent Normal	17	0 0	0 0	17 100
Normal	3	0 0	0 0	3 100

## Data Availability

All data generated or analyzed during this study are included in this published article.

## Supplementary Materials

The following supporting information can be downloaded at: https://www.mdpi.com/article/10.3390/cells14020128/s1, Table S1: Details of Primary/Secondary Antibodies.
