# Supplementary material for: Association of Structural Maintenance of Chromosome-1A Phosphorylation with Progression of Breast Cancer
Source: Cells. 2025 Jan 17;14(2):128. doi: 10.3390/cells14020128 (PMC11764376; doi:10.3390/cells14020128)
Supplement: Supplementary file 1 [file cells-14-00128-s001.zip › cells-3310965-supplementary.pdf]

**Supplemental Table S1**

**Details of Primary/Secondary Antibodies.**

| Antibodies | Source | Identifier |
|---|---|---|
| Rabbit anti-SMC1 | Bethyl Labs | Cat # A300-050A |
| Rabbit anti-pSMC1 (phosphor-Ser 966) | Bethyl Labs | Cat # A300-055A |
| Rabbit anti-CD44 | Cell Signaling | Cat # 3578 |
| Rabbit anti-N-cadherin | Cell Signaling | Cat # 13116 |
| Rabbit anti-GAPDH | Cell Signaling | Cat # 5174 |
| Rabbit Histone H3 (D1H2) mAb | Cell Signaling | Cat # 4499 |
| Rabbit E-Cadherin | Cell Signaling | Cat # 3195 |
| Goat anti-Rabbit-HRP | Thermo Scientific | Cat # 31460 |
| Rabbit anti-mouse-HRP | Thermo Scientific | Cat # 31452 |
| Goat anti-Rabbit-Dylight-488 conjugated | Bethyl Labs | Cat # A50-309D3 |
| Goat anti-Rabbit-Dylight-550 conjugated | Bethyl Labs | Cat # A50-309D3 |
| IRDye® 800CW Goat anti-Rabbit Secondary antibody | LICOR Biotechnology | [P/N 926-32211] |
| CellTag 700 Stain | LICOR Biotechnology | [P/N 926-41090] |
